# Epidemiology of Hypertension in Bangladeshi People with Type 2 Diabetes Attending an Urban Healthcare Center: Impact of the 2017 American College of Cardiology (ACC)/American Heart Association (AHA) Hypertension Clinical Practice Guidelines

**DOI:** 10.7759/cureus.90839

**Published:** 2025-08-23

**Authors:** Md Rakibul-Hasan, Kaniz Fatema Yeasna, Choman Abdullah Mohana

**Affiliations:** 1 Endocrinology and Metabolism, Medical College for Women and Hospital, Dhaka, BGD; 2 Endocrinology and Metabolism, Mymensing Medical College Hospital, Mymensing, BGD

**Keywords:** bangladesh, blood pressure, epidemiology, hypertension, type 2 diabetes

## Abstract

Background

The American College of Cardiology (ACC) and the American Heart Association (AHA) lowered the threshold for defining hypertension (HTN) and for control status in 2017. The effect of the new cutoff on the prevalence of HTN and its burden among Bangladeshi people with type 2 diabetes mellitus (T2DM) has not been well studied. This study aimed to assess the prevalence and predictors of HTN, along with the blood pressure (BP) control status among Bangladeshi people with T2DM using the traditional cutoff (HTN: systolic BP (SBP) ≥140 mmHg and/or diastolic BP (DBP) ≥90 mmHg, controlled HTN: <140 and <90 mmHg, respectively) and to see the impact of 2017 ACC/AHA cutoff (HTN: SBP ≥130 mmHg or DBP ≥80 mmHg, controlled HTN: <130 and <80 mmHg, respectively) on the burden of HTN.

Methodology

This cross-sectional study utilized retrospective data from electronic medical records at a single urban healthcare center from August 2022 to April 2025. Patients with T2DM, aged 18 years or older, were included through a consecutive sampling method. Clinical and demographic information was collected from previous medical records. Patients with gestational diabetes mellitus (DM), T1DM, secondary DM, or T2DM with pregnancy or lactation were excluded. BP was measured using standardized sphygmomanometers under ideal conditions. All abnormal BP readings were cross-checked with repeat measurements at intervals. Data were entered into SPSS (version 25.0; IBM Corp., Armonk, NY) and analyzed. A *P*-value of <0.05 with a 95% confidence interval (CI) was considered significant.

Results

Among 1,444 participants (median age: 50 years, interquartile range (IQR) 40-59; body mass index (BMI): 27.2 kg/m^2^, IQR 24.6-30.2; duration of DM: median 7.0 years, IQR 3.0-13.0; female: 804 (55.7%)), HTN was observed in 929 (64.3%) subjects (previously diagnosed (831, 57.5%), newly diagnosed (98, 6.8%)) using traditional cutoff. Using the 2017 ACC/AHA guideline alarmingly increased the number of newly diagnosed HTN from 98 (6.8%) to 385 (26.7%), raising the total HTN subjects to 1,216 (84.2%). Among previously diagnosed HTN subjects, only 551 (66.3%) were receiving treatment, of whom 286 (51.9%) were maintaining their BP within target ranges, which was reduced to 98 (17.8%) using the 2017 ACC/AHA criteria. Multivariate logistic regression revealed individuals aged 40-59 years had over three times the odds (adjusted odds ratio (AOR): 3.08, 95% CI: 1.87-5.08), and those aged ≥60 had more than six times the odds (AOR: 6.45, 95% CI: 3.04-13.72) of HTN compared to 18-39 years. BMI (23-27.4 kg/m², AOR: 3.64; 95% CI: 1.76-7.50; BMI ≥ 27.5 kg/m², AOR: 3.54; 95% CI: 1.73-7.22 compared to BMI < 23 kg/m²). Poor glycemic control (HbA1c ≥ 7.0%, AOR: 2.01; 95% CI: 1.20-3.35 compared to HbA1c < 7.0%) was independently associated with increased odds of prevalent HTN. However, female sex (*P *= 0.405) and DM duration ≥10 years (*P *= 0.523) were not associated with HTN after adjustment.

Conclusions

Adopting the 2017 ACC/AHA criteria, we observed that roughly four out of five people with T2DM were hypertensive, while only one-fifth had controlled BP.

## Introduction

Hypertension (HTN) and type 2 diabetes mellitus (T2DM) frequently coexist and synergistically contribute to the global burden of cardiovascular disease. In individuals with T2DM, HTN significantly accelerates the development of both microvascular and macrovascular complications, including nephropathy, retinopathy, ischemic heart disease, and stroke [1-3]. Therefore, optimal detection and control of blood pressure (BP) are critical components of diabetes mellitus (DM) management. A nationwide survey from Bangladesh reported the prevalence of diabetes and prediabetes was 9.7% and 22.4%, respectively [4], whereas the prevalence of HTN was found 17.9% in the adult population aged ≥18 years using the traditional cutoff (140/90 mmHg), which was increased to 40.7% using the 2017 ACC/AHA guideline [5]. The Bangladesh Demographic and Health Survey data from 2017-2018 analyzed a total of 12,863 people aged 18 years or older, reporting a prevalence of HTN of 27.4% [6]. In Bangladesh, where the prevalence of T2DM has been increasing steadily over the past decade, HTN remains an underdiagnosed and inadequately managed comorbidity.

Traditionally, HTN has been defined as systolic BP (SBP) ≥140 mmHg or diastolic BP (DBP) ≥90 mmHg according to most of the guidelines [7,8]. However, in 2017, the American College of Cardiology (ACC) and the American Heart Association (AHA) updated the diagnostic threshold to SBP ≥130 mmHg or DBP ≥80 mmHg, reflecting evidence that cardiovascular risk begins to increase at lower BP levels. This change is also supported by the International Society of Hypertension and the European Society of Cardiology/European Society of Hypertension (ESC/ESH) BP guidelines [9]. The American Diabetes Association (ADA) has also endorsed this lower threshold in its 2025 guidelines, particularly emphasizing its relevance in individuals with DM who are at elevated cardiovascular risk [10]. The Systolic BP Intervention Trial (SPRINT) demonstrated that targeting a systolic SBP of <120 mmHg resulted in a 25% reduction in cardiovascular event rates among high-risk individuals without DM [11]. However, individuals with DM were specifically excluded from this trial, limiting its direct applicability to the diabetic population. In contrast, the Strategy of BP Intervention in the Elderly Hypertensive Patients (STEP) trial, which included nearly 20% of participants with DM, found that treating HTN to a target SBP <130 mmHg was associated with a significant reduction in cardiovascular events [12].

Adopting this updated definition is expected to dramatically increase the proportion of individuals classified as hypertensive. Yet, there are limited data on the epidemiological impact of these revised criteria in low- and middle-income countries, particularly among Bangladeshi adults with T2DM. This gap in knowledge is concerning, given the rapid epidemiological transition and growing burden of non-communicable diseases in the region [5,13].

The present study aimed to investigate the prevalence, treatment patterns, and control rates of HTN in a large cohort of Bangladeshi adults with T2DM using both conventional and revised BP definitions according to the 2017 ACC/AHA Hypertension Clinical Practice Guidelines. Additionally, we evaluated the age- and sex-specific impact of the updated cutoff values and identified independent risk factors associated with HTN in this population.

## Materials and methods

Study design and study population

This cross-sectional study enrolled patients from an endocrinology outpatient department of a single private healthcare center in Dhaka, Bangladesh, using the electronic database. All consecutive patients with T2DM, aged ≥ 18 years, irrespective of their duration of DM, were included in the study. We included a total of 1,444 subjects with T2DM from August 2022 to April 2025. Patients’ clinical and demographic information was collected from previous medical records and the most recent investigation reports. Patients with DM other than T2DM (e.g., gestational DM, type 1 DM, secondary DM, or other specific types) and those with T2DM during pregnancy or lactation were excluded from the study. BP was measured using standardized sphygmomanometers. A single investigator measured the BP of all patients while they were seated with the arm at heart level, after at least 5 minutes of rest. All abnormal BP readings were cross-checked by repeat measurement after at least a seven-day interval, including measurement on the opposite arm.

Operational definition

HTN was diagnosed based on the previous medical record of a confirmed diagnosis of HTN or a drug history of taking medicines for HTN. For all new diagnoses of HTN, both the traditional diagnostic cutoff value recommended by the International Society of Hypertension Global Hypertension Practice Guidelines 2020 and Hypertension in Adults: Diagnosis and Management (NICE guideline, 2023) (SBP ≥140 mmHg and/or DBP ≥90 mmHg) [7,8], and the new cutoff value (SBP ≥130 mmHg and/or DBP ≥80 mmHg) recommended by the 2017 ACC/AHA and the American Diabetes Association 2025 [9,10], were used to define HTN. Incidentally, found high BP on a single episode was not considered hypertensive. Repeat measurement of BP on the last follow-up was used to categorize them as either hypertensive or normotensive. A detailed drug history was collected from the patients' previous medical records. The status of BP control was determined based on the last follow-up BP measurement. All hypertensive patients were categorized as having either controlled or uncontrolled BP based on the traditional cutoff (Controlled: SBP <140 mmHg and DBP <90 mmHg; Uncontrolled: SBP ≥140 mmHg and/or DBP ≥90 mmHg) and the 2017 ACC/AHA cutoff (Controlled: SBP <130 mmHg and DBP <80 mmHg; Uncontrolled: SBP ≥130 mmHg and/or DBP ≥80 mmHg). Body mass index (BMI) was calculated by measuring weight in kilograms divided by height in meters squared. The BMI was categorized as underweight (BMI < 18.50 kg/m^2^), normal (BMI 18.5-22.9 kg/m^2^), overweight (23.00 to <27.5 kg/m^2^), and obese (≥27.5 kg/m^2^) [14]. The status of glycemic control was defined based on the last recorded glycated hemoglobin (HbA1c) value. Glycemic status was categorized as controlled DM if HbA1c was <7.0% and uncontrolled DM if it was ≥7.0%, according to the American Diabetes Association 2025 guideline [15].

Ethical approval

The study was approved by the Ethical Committee of the Medical College for Women and Hospital, Uttara Model Town, Dhaka, Bangladesh (memo number: MCWH/Ethical Committee/2025/16(4)).

Statistical analysis

Data distribution was checked using the Kolmogorov-Smirnov and Shapiro-Wilk tests. Continuous variables were expressed as mean ± standard deviation (SD) or as median with interquartile range (IQR), depending on the data distribution. Categorical variables were presented as frequencies (numbers) and percentages. An independent sample t-test or Mann-Whitney U test was used to compare quantitative variables between the two groups. A binary logistic regression was used to find out the predictors or risk factors for HTN among the study subjects.

## Results

The baseline characteristics of the study participants are presented in Table 1. We observed patients with HTN were elderly (median age: 53 vs. 42 years, *P *< 0.001), with higher BMI (27.4 kg/m^2^ vs. 27.0 kg/m^2^, *P *= 0.036), longer duration of DM (≥10 years vs. <10 years: 336 (46.5%) vs. 118 (28.4%), *P* < 0.001; *n *= 1,138), associated with more HTN-related complications and comorbidities (chronic kidney disease (CKD): 71 (7.6%) vs. 12 (2.3%), *P* < 0.001; ischemic heart disease (IHD): 117 (12.6%) vs. 26 (5.0%), *P* < 0.001; dyslipidemia: 585 (63.0%) vs. 179 (34.8%), *P* < 0.001). A higher number of patients in the HTN group were receiving treatment with insulin (326 (35.7%) vs. 135 (27.4%), *P* < 0.001), with poor glycemic control (HbA1c ≥ 7%: 559 (83.7%) vs. 272 (77.5%), *P *= 0.015; *n *= 1,019) (Table 1).

**Table 1:** Demographic and clinical characteristics of the study participants based on the presence or absence of hypertension (n = 1,444). Percentages within the parentheses are over the column total. HTN is defined as SBP ≥ 140 mmHg and/or DBP ≥ 90 mmHg. Comparison of qualitative variables (age, SBP, DBP, and BMI) between two groups was done by the Mann-Whitney U test due to the skewed distribution of data, whereas for qualitative variables, the chi-square test was used. HTN, hypertension; BP, blood pressure; SBP, systolic BP; DBP, diastolic BP; BMI, body mass index; HbA1c, glycated hemoglobin (%); IQR, interquartile range; OAD, oral anti-diabetic drug; CKD, chronic kidney disease; IHD, ischemic heart disease

Variable	Total (*n* = 1,444)	Normotensive (*n* = 515)	Hypertensive (*n* = 929)	*P*-value
Age (median with IQR)	50 (40-59)	42 (35-52)	53 (44.5-62.0)	<0.001
Age category (years)				
18-39	311 (21.5)	194 (37.7)	117 (12.6)	<0.001
40-59	773 (53.5)	263 (51.1)	510 (54.9)	
≥60	360 (25.0)	58 (11.3)	302 (32.5)	
Gender				
Female	804 (55.7)	256 (49.7)	548 (59.0)	0.001
Male	640 (44.3)	259 (50.3)	381 (41.0)	
SBP (mmHg), median with IQR	130 (120-140)	120 (110-130)	138 (120-150)	<0.001
DBP (mmHg), median with IQR	80 (70-82)	80 (70-80)	80 (70-85)	<0.001
BMI (kg/m^2^), median with IQR, *n* = 692	27.2 (24.6-30.2)	27.0 (24.5-29.7)	27.4 (24.9-30.6)	0.036
BMI (kg/m^2^) category (*n* = 692)				
<23	78 (11.3)	44 (15.9)	34 (8.2)	0.007
23 to <27.5	282 (40.8)	108 (39.1)	174 (41.8)	
≥27.5	332 (48.0)	124 (44.9)	208 (50.0)	
Duration of DM (*n* = 1138)				
<10 years	684 (60.1)	297 (71.6%)	387 (53.5)	<0.001
≥10 years	454 (39.9)	118 (28.4)	336 (46.5)	
Mode of Rx of DM				
OAD	1142 (81.2)	386 (78.5)	756 (82.7)	0.051
Insulin	461 (32.8)	135 (27.4)	326 (35.7)	0.002
OAD with insulin	360 (25.6)	103 (20.9)	257 (28.1)	0.003
Status of HbA1c (*n* = 1,019)				
<7.0%	188 (18.4)	79 (22.5)	109 (16.3)	0.015
≥7.0%	831 (91.6)	272 (77.5)	559 (83.7)	
Comorbidities				
CKD	83 (5.7)	12 (2.3)	71 (7.6)	<0.001
IHD	143 (9.9)	26 (5.0)	117 (12.6)	<0.001
Dyslipidemia	764 (52.9)	179 (34.8)	585 (63.0)	<0.001

Prevalence and control of HTN according to the 2017 ACC/AHA guidelines

Using the traditional cutoff value, we observed that 929 (64.3%) subjects were hypertensive, among whom 98 (6.8%) were newly diagnosed. Newly diagnosed HTN accounted for 10.57% (98 of 929) of the total hypertensive patients. Using the 2017 ACC/AHA guidelines, alarming increased the number of newly diagnosed HTN cases from 98 to 385 (6.8%-26.7%), raising the prevalence of HTN to 84.2% (1,216 of 1,444) among the study participants. Among previously diagnosed cases of hypertensive patients, only 551 (66.3%) were receiving treatment to control BP, of whom only 286 (51.9%) were maintaining their BP within target ranges, which was reduced to only 98 (17.8%) if we use the 2017 ACC/AHA targets. The majority of patients with uncontrolled HTN had uncontrolled SBP (463, 88.7%), followed by uncontrolled DBP (223, 42.7%) and uncontrolled both SBP and DBP (164, 31.4%). Using the 2017 ACC/AHA guidelines, the uncontrolled DBP was the most prevalent (902, 84.6%), followed by uncontrolled SBP (772, 72.4%) and uncontrolled both SBP and DBP (608, 57.0%) (Table 2).

**Table 2:** Impact of AHA 2017 new BP cutoff (130/80 mmHg) on hypertension prevalence, and BP control status among the study participants (n = 1444). Within the parentheses, percentages are expressed over the column total. Group A: HTN is defined as SBP ≥ 140 and/or DBP ≥ 90 mmHg. BP controlled is defined as SBP < 140 mmHg with DBP < 90 mmHg. Group B: HTN is defined as SBP ≥ 130 and/or DBP ≥ 80 mmHg. BP controlled is defined as SBP < 130 mmHg with DBP < 80 mmHg. Uncontrolled SBP, DBP, and both BP are not mutually exclusive. HTN, hypertension; BP, blood pressure; HTN, hypertension; SBP, systolic BP; DBP, diastolic BP; AHA, American Heart Association

Characteristics	Group A	Group B
Cutoff	140/90	130/80
Prevalence of HTN (*n *= 1,444)		
Previously diagnosed HTN	831 (57.5)	831 (57.5)
Newly diagnosed HTN	98 (6.8)	385 (26.7)
Total	929 (64.3)	1216 (84.2)
Patient receiving treatment among previously diagnosed cases (*n *= 831)	551 (66.3)	551 (66.3)
BP control status among all hypertensive patients	*n *= 929	*n *= 1,216
Controlled	407 (43.8)	150 (12.3)
Uncontrolled	522 (56.2)	1,066 (87.7)
BP control status among previously diagnosed hypertensive patients (*n *= 831)		
Controlled	404 (48.6)	150 (18.1)
Uncontrolled	427 (51.4)	681 (81.9)
BP controlled status among hypertensive patients receiving treatment (*n *= 551)		
Controlled	286 (51.9)	98 (17.8)
Uncontrolled	265 (48.1)	453 (82.2)
Types of uncontrolled HTN among all uncontrolled HTN patients	*n* = 522	*n *= 1,066
Uncontrolled SBP	463 (88.7)	772 (72.4)
Uncontrolled DBP	223 (42.7)	902 (84.6)
Uncontrolled both BP	164 (31.4)	608 (57.0)

Age- and sex-specific prevalence of HTN according to the 2017 ACC/AHA guidelines

HTN prevalence increased substantially across all age and sex groups using the 2017 ACC/AHA new cutoff. Among females, the number of hypertensive subjects rose from 548 (68.2%) to 684 (85.1%) out of 804 subjects, and among males, from 381 (59.5%) to 532 (83.1%) out of 640. The increment was particularly notable among younger adults (18-39 years age group), prevalence nearly doubled in both males and females. In older adults (≥60 years), almost 90% of both males and females had been identified as hypertensive (Table 3). Significant increase in the prevalence of HTN in parallel with the increasing age of the study participants for both male and female (*P* < 0.001). No sex-specific difference in HTN prevalence was seen between males and females using the 2017 ACC/AHA guidelines. Still, the difference was maintained with female predominance after 40 years of age using the traditional cutoff.

**Table 3:** Impact of AHA 2017 new blood pressure cutoff on hypertension prevalence at the different age and sex categories (n = 1,444). *P-values among different age groups within the same sex category. Group A: HTN is defined as SBP ≥ 140 mmHg and/or DBP ≥ 90 mmHg. Group B: HTN is defined as SBP ≥ 130 mmHg and/or DBP ≥ 80 mmHg. Comparison between the two groups and within groups was done using the chi-square test.

Characteristics	Group A (≥140/90)		*P*-value	Group B (≥130/80)		*P*-value
Age group (year)	Female (*n *= 804)	Male (*n *= 640)		Female (*n *= 804)	Male (*n *= 640)	
All (*n *= 1444)	548 (68.2)	381 (59.5)	0.001	684 (85.1)	532 (83.1)	0.313
18-39 (*n *= 311)	68 (38.6)	49 (36.3)	0.673	134 (76.1)	100 (74.1)	0.676
40-59 (*n *= 773)	303 (70.8)	207 (60.0)	0.002	366 (85.5)	289 (83.8)	0.502
≥60 (*n *= 360)	177 (88.5)	125 (78.1)	0.008	184 (92.0)	143 (89.4)	0.391
*P-value	<0.001	<0.001		<0.001	0.002	

Antihypertensive medication prescription patterns

Among the 551 hypertensive subjects receiving treatment, the majority of them (254, 46.1%) were getting dual therapy, only 169 (30.7%) were getting a single pill, and 128 (23.2%) were receiving three or more drugs. ACE inhibitors or ARBs were the most commonly prescribed anti-hypertensive drugs (430 out of 551, 78.0%), which aligned with the recommendations of the majority of the guidelines. Calcium channel blockers (CCBs) were prescribed in 347 (63.0%) of the patients, followed by beta-blockers in 203 (36.8%), diuretics in 85 (15.4%), and alpha-blockers in 21 (3.8%) (Table 4).

**Table 4:** Anti-hypertensive medication prescription patterns among the hypertensive patients receiving treatment (n = 551). Within the parentheses, percentages are expressed over the column total. ACEi, angiotensin converting enzyme inhibitors; ARB, angiotensin II receptor blocker

Characteristics	Frequency (%)
Number of anti-hypertensive medications prescribed (*n* = 551)	
One drug	169 (30.7)
Two drugs	254 (46.1)
≥Three drugs	128 (23.2)
Types of anti-hypertensive medication prescribed (*n *= 551)	
ACEi or ARBs	430 (78.0)
Calcium channel blockers	347 (63.0)
Beta-blockers	203 (36.8)
Diuretics	85 (15.4)
Alpha blockers	21 (3.8)

Risk factors for hypertension

Multivariate logistic regression identified several independent risk factors for HTN. Compared to those aged 18-39 years, individuals aged 40-59 years had over three times the odds (AOR: 3.08, 95% CI: 1.87-5.08), and those aged ≥60 had more than six times the odds (AOR: 6.45, 95% CI: 3.04-13.72) of HTN. Overweight and obesity were also strongly associated with HTN: BMI 23-27.4 kg/m² (AOR: 3.64; 95% CI: 1.76-7.50) and BMI ≥27.5 kg/m² (AOR: 3.54; 95% CI: 1.73-7.22). Poor glycemic control (HbA1c ≥ 7.0%) was independently associated with the risk of HTN (AOR: 2.01; 95% CI: 1.20-3.35). However, female sex and DM duration ≥10 years were not independently associated with HTN after adjustment (Table 5).

**Table 5:** Multivariate binary logistic regression analysis to assess the risk factors for hypertension in T2DM. Here, hypertension is defined as SBP ≥ 140 and/or DBP ≥ 90 mmHg. DM controlled, HbA1c < 7.0%, uncontrolled HbA1c ≥ 7.0%. Variables in the equation for regression: age category, sex, BMI category, duration of DM, status of glycemic control (samples included in the analysis = 427). OR, odds ratio; AOR, adjusted odds ratio; CI, confidence interval; BMI, body mass index; HbA1c, glycated hemoglobin (%); T2DM, type 2 diabetes mellitus

Variables	OR (95% CI)	*P*-value	AOR (95% CI)	*P*-value
Gender: Male (Ref)	1		1	
Female	1.45 (1.17-1.81)	0.001	1.20 (0.78-1.84)	0.405
Age (year): 18-39 (Ref)	1		1	
40-59	3.21 (2.44-4.22)	<0.001	3.08 (1.87-5.08)	<0.001
≥60	8.63 (6.00-12.41)	<0.001	6.45 (3.04-13.72)	<0.001
BMI (kg/m^2^): <23.0 (Ref)	1		1	
>23.0 to 27.4	2.08 (1.25-3.46)	0.005	3.64 (1.76-7.50)	<0.001
≥27.5	2.17 (1.32-3.58)	0.002	3.54 (1.73-7.22)	0.001
DM duration (year): <10 (Ref)	1		1	
≥10	2.18 (1.68-2.83)	<0.001	0.85 (0.52-1.40)	0.523
HbA1c: <7.0 (Ref)	1		1	
≥7.0	1.49 (1.10-2.06)	0.016	2.01 (1.20-3.35)	0.008

## Discussion

This study provides a comprehensive insight into the epidemiology of HTN among Bangladeshi adults with T2DM, using both the traditional cutoff (per ISH 2020 and NICE 2023 guidelines) and the 2017 ACC/AHA guidelines. Our findings reveal an alarmingly high prevalence of HTN in this population, particularly when the updated threshold of the 2017 ACC/AHA is applied, with substantial implications for clinical management and public health policy.

HTN prevalence and impact of the 2017 ACC/AHA revised cutoff

Using the traditional cutoff, the prevalence of HTN was 64.3%, consistent with regional estimates ranging from 60%-70% in diabetic populations [2,5]. However, application of the 2017 AHA/ACC and ADA 2025 guideline recommendations increased the prevalence to 84.2%, with over a quarter (26.7%) of cases newly identified. This dramatic increase parallels global trends, where adoption of the updated definition significantly expanded the hypertensive population [9,10,16]. An Indian study, which included general people, published in 2020, found that 70.9% of subjects were classified as hypertensive using the 2017 ACC/AHA guidelines, compared to only 38.5% when using the traditional cutoff [17]. HTN prevalence in patients with DM in the United States was observed in 76.3% and 66.0% for adult women and men, respectively, according to the 2017 ACC/AHA and ADA recommendations [18]. These study findings mirror the pattern observed in our study. Such a shift holds particular importance for Bangladesh, where the dual burden of DM and HTN is growing rapidly amidst limited healthcare resources. According to the Bangladesh Demographic and Health Survey (BDHS) 2017-2018, approximately 20% of adults have HTN, with only 44% aware of their condition and just 14% having controlled BP [6]. In diabetic populations, this burden is amplified, and our study adds new evidence showing that nearly 9 in 10 older adults with T2DM now meet the hypertensive criteria under the 2017 ACC/AHA guidelines.

Age- and sex-specific differences

The 2017 ACC/AHA guidelines particularly impacted younger adults (18-39 years), with prevalence nearly doubling. This is concerning, as early-onset HTN in T2DM carries a cumulative risk for cardiovascular and renal outcomes over time [1]. While a female predominance in HTN was observed under the traditional definition, the sex disparity disappeared with the revised criteria, suggesting that cardiovascular risk at lower thresholds may be more evenly distributed across genders. However, this pattern is not universally observed. Other studies have reported a persistent male predominance in HTN even under the revised definition, possibly due to differences in study populations, lifestyle factors, or health-seeking behavior [16,17].

Antihypertensive treatment patterns

In our study, 66.3% hypertensive patients were receiving treatment, which is relatively better than the global trend, where 47% of women and 38% of men were receiving treatment for HTN [19]. The most commonly prescribed antihypertensive class was ACE inhibitors or ARBs (78.0%), followed by calcium channel blockers (63.0%), in line with current guideline recommendations [7,10]. However, only 30.7% of patients were managed with monotherapy, while nearly one-quarter required three or more agents. This polypharmacy reflects the complex interplay between DM and HTN. It also highlights the need for personalized, evidence-based regimens and consideration of patient adherence and cost barriers.

BP control status

Globally, the BP control rates among people with HTN in 2019 were 23% for women and 18% for men using the traditional cutoff [19]. BP control status in our population is relatively better than the global trend, and we observed that nearly 44% of our all-hypertensive patients have their BP under control with the same cutoff. The control status sharply dropped to 17.8% using the 2017 ACC/AHA targets. The BP control status is not satisfactory worldwide, even among developed countries. Only one-fifth of the hypertensive population had their BP controlled to less than 130/80 mm Hg between 2021 and 2023 [19,20]. This indicates significant therapeutic inertia or suboptimal treatment intensification-a well-documented issue in diabetes care [3,9]. Our findings align with previous studies suggesting that systolic SBP is the primary contributor to uncontrolled HTN. In our cohort, uncontrolled SBP was found in 88.7% of hypertensives under the traditional cutoff. These results underscore the need for aggressive SBP management, especially considering its well-established role in cardiovascular risk among older adults [11,12].

Risk factors

Multivariate analysis identified older age, higher BMI, and poor glycemic control as significant independent risk factors for HTN. Individuals aged ≥60 years had more than six times the odds of being hypertensive compared to those aged 18-39 years, consistent with vascular ageing and arterial stiffness [3,4]. Similarly, overweight and obese individuals (BMI ≥ 23 kg/m²) had three to four times higher odds of HTN. Poor glycemic control (HbA1c ≥ 7.0%) also showed a twofold increased risk, supporting the interrelationship between hyperglycemia and endothelial dysfunction [2]. Interestingly, female sex and duration of DM were not independently associated with HTN in adjusted analysis, contrasting with some studies that have identified chronic hyperglycemia duration as a risk factor [5]. These differences may reflect variations in study design, population characteristics, and definitions of exposure.

Implications and future directions

The updated 2017 ACC/AHA guidelines substantially increase the number of individuals classified as hypertensive, posing both opportunities and challenges. While it facilitates earlier detection and potential prevention of cardiovascular events, it also underscores the urgent need to enhance treatment intensity and healthcare system readiness in low-resource settings such as Bangladesh. Community-based screening programs, healthcare provider training, better medication access, and interventions targeting obesity and glycemic control are critical. Longitudinal studies are needed to assess whether earlier diagnosis and tighter BP control ultimately lead to improved cardiovascular outcomes in the Bangladeshi diabetic population.

This study is limited by its study design using retrospective data from a single urban center, which may not fully represent the rural or national population. Additionally, reliance on clinical records may lead to underestimation or misclassification of BP control status. Medication adherence for chronic disease is very poor, which was not checked in our study, which could influence the overall result of the control status of BP among hypertensive patients. Many factors directly or indirectly increase the risk of HTN (e.g., smoking, physical activities, dietary salt intake), which were not included during regression due to study design. These factors may be included in the cross-sectional study, which would give more accurate information. Despite these limitations, the study findings give us some epidemiological information regarding the burden of uncontrolled HTN in an urban setting.

## Conclusions

In conclusion, HTN is highly prevalent among Bangladeshi adults with T2DM, and the adoption of the updated 2017 ACC/AHA guidelines and ADA 2025 recommendations further amplifies this burden. A large portion of the population with uncontrolled diabetes and HTN poses a great challenge to our healthcare system by increasing cardiovascular complications. A large-scale multicenter cross-sectional study from different divisions of Bangladesh should be planned to quantify the problem in the real-world setting. A long-term prospective follow-up study regarding the implications of the new cutoff value of the 2017 ACC/AHA guideline and recommendation from the ADA 2025 guideline may be considered before generalizing it for all patients with T2DM in our setting.
